# The correlation between inflammatory markers and postoperative pulmonary infection in patients with aneurysmal subarachnoid hemorrhage and the construction of prediction model

**DOI:** 10.3389/fneur.2025.1563106

**Published:** 2025-06-24

**Authors:** Hongxiang Chen, Fangyu Yang, Yulong Zhao, Jiaming Liu, Yichun Tang, Shunyao Du, Zezheng Fan, Hongge Fan, Penglin Ren, Xu Gao

**Affiliations:** Department of Neurosurgery, The General Hospital of Northern Theater Command, Shenyang, Liaoning, China

**Keywords:** aneurysmal subarachnoid hemorrhage, neuroinflammation, pneumonia, inflammatory markers, nomogram, predictive model

## Abstract

**Background:**

Aneurysmal subarachnoid hemorrhage (aSAH)-related pneumonia has a high incidence, with a significant impact on prognosis. This study aims to investigate the correlation between inflammation markers and aSAH-related pneumonia and develop a clinical prediction model for aSAH-related pneumonia using inflammation markers for better clinical decisions.

**Method:**

In this retrospective study, we retrieved data including demographic, imaging, laboratory, and clinical complications of patients with aSAH admitted to the General Hospital of Northern Theater Command between January 2018 and January 2024. Multiple logistic regression models were employed to perform data analysis.

**Results:**

The results revealed that 226 patients had pneumonia. In the ROC curve analysis, LDH had higher predictive accuracy than other biomarkers. The optimal cut-off value for predicting pneumonia using LDH (Lactate dehydrogenase) was 1.545, with a sensitivity of 73% and a specificity of 81.5%. The relevant risk factors identified by multivariate logistic regression were incorporated into the Nomogram. The calibration curve showed a strong agreement between the predicted and observed probabilities. The C index was 0.82, the ROC curve demonstrated excellent discrimination. Besides, the AUC for predicting pneumonia using the Nomogram was 0.82 (95% CI 0.791–0.823; *p* < 0.001).

**Conclusion:**

In summary, inflammatory markers have a certain predictive value for pneumonia in aSAH patients, with LDH being significantly linked to pneumonia after aSAH with excellent individual predictive capacity. The concordance between the predicted and observed probabilities is strong, confirming an excellent predictive ability for pneumonia following aSAH.

## 1 Introduction

Subarachnoid hemorrhage (SAH) is a severe and complex devastating disease with a 30-day mortality rate ranging from 18 to 40% (1, 2). While acknowledging better progress in treatment strategies and postoperative care plans for SAH, recent investigations have shown that the postoperative mortality rate for patients with aneurysms still stands at 20% (3). This indicates the need to explore the incidence of postoperative complications in aSAH patients. For example, some studies have shown that the incidence of hydrocephalus is 6.5–85% (4). Others have reported an incidence of delayed cerebral ischemia of 20–30% (5). Pneumonia has been reported following aSAH, with an incidence of 28–30% (6). These represent alarming statistics, as the incidence may be higher than reported. The current diagnosis of aSAH-related pneumonia is still primarily based on clinical presentation, vital signs, and imaging studies. This may result in delayed treatment and missing the optimal treatment window. These clinical hurdles require corresponding markers to improve the capacity to predict, diagnose, and monitor clinical deterioration. Recent retrospective investigations have also shown that the level of neuroinflammation after hemorrhagic stroke dictates the occurrence of aSAH-related pneumonia (7). More inflammatory markers are currently available, yet a systematic description of their efficacy of inflammatory markers is lacking. Besides, there is still no comprehensive clinical prediction model for aSAH-related pneumonia. Therefore, more reliable and easily measurable markers for aSAH-related pneumonia will help in early prediction and risk stratification, as well as provide clues for further research on preventive antibiotic treatment. This work evaluates the efficacy of inflammatory marker tests and develops an inflammatory marker model for predicting aSAH-related pneumonia.

Neuroinflammation is an important factor in the pathophysiology of aSAH, and a cause of brain damage in the acute and chronic phases of SAH. Based on previous studies, the secondary neuroinflammatory response after aSAH has numerous features, including the release of inflammatory mediators, immune cell activation, blood–brain barrier disruption, and neuronal apoptosis, which are associated with the activation and involvement of multiple signaling pathways (8). Notably, aSAH can cause neutrophil infiltration at the site of injury and increase systemic neutrophil count by improving IL-6 (9). This process stimulates neutrophils to migrate to the brain within the first 12–48 h after aSAH. Additionally, microglia activation and the recruitment of monocytes are easily evident within the first 48 h of aSAH, exacerbating neuroinflammation (10). Neutrophil accumulation on the endothelial membrane increases oxidative stress (through myeloperoxidase) and lipid peroxidation, resulting in endothelial injury (9, 11, 12). Toll-like receptor 4 (TLR4) becomes enhanced in activated microglia and macrophages, resulting in the secretion of TNF (13). The free heme formed from red cell hemolysis produces reactive oxygen species, causing MMP-9 upregulation and destruction of the vascular basement membrane (14). Therefore, this study aims to investigate whether the changes in peripheral blood inflammatory markers caused by the activation of neuroinflammation mechanisms after aSAH could predict the risk of aSAH-induced pneumonia. We also seek to understand the independent and joint correlations of various inflammatory markers. The term “pneumonia linked to aSAH” used in this study specifically refers to pneumonia resulting from neuroinflammation-induced mechanisms. This definition explicitly excludes pneumonia attributable to aspiration, prolonged bed rest (hypostatic pneumonia), or mechanical ventilation (ventilator-associated pneumonia, VAP).

## 2 Materials and methods

### 2.1 Patients

**Figure 1:**
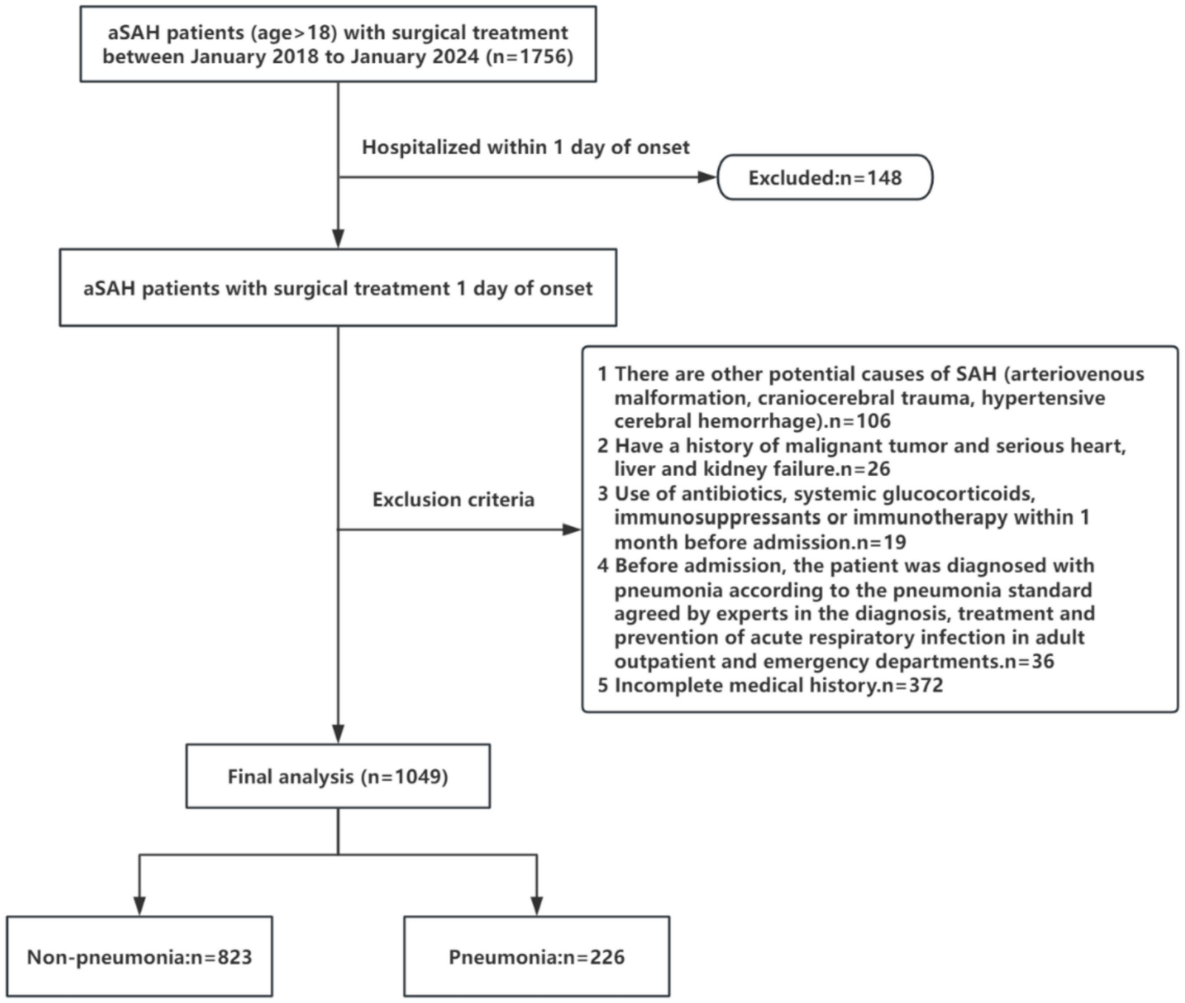
Flow chart of Patients of the study. aSAH, aneurysmal subarachnoid hemorrhage.

Patients diagnosed with aSAH admitted to the General Hospital of Northern Theater Command were recruited between January 2018 and January 2024. This study adhered to the ethical committee and Helsinki Declaration requirements. The inclusion criteria for patients included: (1) patients aged 18 years or above, first episode of SAH, and admission within 24 h of symptom onset and blood sample collection within 24 h of admission; (2) diagnosis of aSAH confirmed by computerized tomography (CT), computed tomography angiography (CTA), or digital subtraction angiography (DSA); (3) underwent surgical clipping of intracranial aneurysm or endovascular treatment;(4) postoperative pulmonary CT scans were performed within 24 h. The exclusion criteria included: (1) other potential causes of SAH (arteriovenous malformations, craniocerebral trauma, hypertension cerebral hemorrhage); (2) history of malignant tumor, severe heart, liver, or kidney dysfunction; (3) use of antibiotics, systemic glucocorticoids, immunosuppressants, or immunotherapy within the last month before admission(These treatment modalities could induce modifications in peripheral immune responses, potentially obscuring the clinical relevance of inflammatory biomarkers.); (4) confirmed pneumonia based on the adult emergency department acute respiratory infection diagnosis and prevention expert consensus within the last month before admission; (5) incomplete medical history; (6) patients receiving conservative (non-surgical) management and those requiring re-intervention for postoperative rebleeding (Figure 1).

### 2.2 Diagnostic criteria for pneumonia

The diagnosis of pneumonia was based on examination by our imaging department suggesting an inflammatory infiltrate in the lung lobes visible on chest CT scan. Some scholars (15) define ventilator-associated pneumonia (VAP) as a new-onset pneumonia occurring ≥48 h after endotracheal intubation—recognized as the most prevalent and lethal nosocomial infection in critical care—our study specifically excluded VAP cases. Given that all patients in our institution underwent aneurysm surgery under general anesthesia with ventilator support, we required pulmonary CT scans to be completed within 24 h postoperatively to rigorously exclude ventilator-related pneumonia. Consequently, our diagnostic time window for pneumonia was strictly defined as occurring within 24 h post-surgery. The reference to hypostatic pneumonia serves to minimize confounding effects, as extended immobilization (prolonged bed rest) may independently elevate pneumonia risk. Including such cases would introduce selection bias, as hypostatic pneumonia represents a mechanistically distinct entity from the acute pneumonia phenotypes under investigation. To rigorously control this confounder, we recalibrated the diagnostic window to 24 h postoperatively—a methodological refinement explicitly designed to exclude immobilization-related pneumonia while preserving the integrity of outcome ascertainment.

### 2.3 Data collection

We obtained data on demographic, imaging, laboratory, and clinical complications of patients with aSAH admitted at the Northern Theater Command General Hospital between January 2018 and January 2024, including 1,049 patients who met the inclusion criteria and passed the neurological examination. The patients were into two groups based on whether they developed related pneumonia after disease onset, i.e., (1) the non-pneumonia group comprising 823 patients, and (2) the pneumonia group comprising 226 patients. The collected general information included: demographic data (age, gender), past medical history (hypertension, diabetes, hyperlipidemia, coronary heart disease, cerebral infarction history, smoking history, alcohol consumption history), general condition (body mass index), admission status (admission heart rate, admission respiratory rate, admission mean arterial pressure, Hunt-Hess score, mechanical ventilation, dysphagia), neuroimaging data (modified Fisher grade, aneurysm location), and laboratory data obtained within 24 h of admission (white blood cell count, neutrophil count, lymphocyte count, monocyte count, NLR, PLR, MLR, SII, SIRI, AISI, CLR, NAR, FAR, red blood cell count, hemoglobin, platelet count, C-reactive protein, prothrombin time, activated partial thromboplastin time, fibrinogen, D-dimer, plasma fibrin degradation product, anticoagulant, lactate dehydrogenase, serum total bilirubin, serum direct bilirubin, AST, ALT, urea, creatinine, glucose, potassium, sodium, chloride, albumin, creatine kinase isoenzyme, B-type natriuretic peptide, cardiac troponin I). We also gathered patient information on complications (cerebral cardiac syndrome, delayed cerebral ischemia, seizures, acute hydrocephalus).

### 2.4 Sample size calculation

Regarding the sample size issue, when calculating the sample size, it was based on studying the factors influencing aSAH-associated pneumonia, identifying independent risk factors by constructing a logistic regression model. The sample size estimation followed the four-step method proposed by Richard Riley et al. (16). According to previous studies the incidence of aSAH-associated pneumonia ranges approximately from 13.2 to 30.8% (17–21). Thus, the positive event rate was set at 30%. With 14 candidate factors to be included in the multivariate model, and based on the incidence rate and the maximum Cox-Snell R-squared (CS R^2^) value, the estimated CS R^2^ value for this study was 0.11. After calculation, the minimum required sample size was determined to be 921 cases. The critical step for ensuring an adequate sample size was Step 3 of the method, with the formula as follows:


$$n=\frac{P}{\left(S-1)\ln(1-\frac{R_{\mathrm{CS}}^{2}}{S}\right)}$$

When the shrinkage factor (S) is set to 0.9 and P presents the number of predictors, the calculated required sample size is 921 cases.

### 2.5 Data analysis

Continuous variables were described using the median with quartiles or the average with standard deviation, whereas categorical data were presented by proportions. The chi-square (χ^2^) test or Fisher’s exact test was used to analyze categorical variables, whereas the Mann–Whitney U test is used to evaluate continuous variables without assuming normality. All variables were analyzed using univariate logistic regression, and variables with *p* < 0.05 were included in multivariate logistic regression. A receiver operating characteristic (ROC) curve, C index, and calibration plot were used to estimate the risk of pneumonia based on a multivariable logistic regression model; its performance was evaluated by ROC analysis, C index, and calibration plot. Internal validation of the nomogram was performed using the Bootstrap method to evaluate overfitting risks. Subgroup and sensitivity analyses were conducted to account for potential confounding factors. Significant differential inflammation markers were analyzed using two analytical models, i.e., the first model was unadjusted, whereas the second model included all relevant confounding factors through a multivariable logistic regression. The area under the ROC curve (AUC) was used to evaluate the predictive accuracy of LDH, SIRI, SII, NLR, and MLR for pneumonia. The optimal cutoff point was determined by the Youden index. Further, the DeLong test was used to compare ROC curves. We also computed adjusted odds ratios (OR) and 95% confidence intervals (CI). Furthermore, the dose–response relationship between LDH, SIRI, SII, PLR, NLR, and MLR and the risk of pneumonia was studied using the restricted cubic splines (RCS) within a logistic regression framework, with four nodes located at the 25, 50, 75, and 95th percentiles, and adjusted for potential confounders. All statistical analyses were performed using R software version 3.6.3 (R Foundation for Statistical Computing, Vienna, Austria). A two-tailed *p*-value < 0.05 was considered statistically significant.

## 3 Results

### 3.1 Results of general information

A total of 1,049 consecutive patients were included in this study, with a median age of 58 years (interquartile range [IQR]: 51–66 years), of whom 407 (49%) were male. The median (IQR) values for the collected biomarkers were as follows: SIRI, 3.98 (2.16–6.94); SII, 1,923.64 (1,094.80–3,150.00); NLR, 9.00 (5.07–14.38); PLR, 195.00 (137.33–294.00); MLR, 0.42 (0.29–0.64); and LDH, 191.50 (163.00–240.00). Among the participants, 226 (21.54%) were diagnosed with pneumonia. Patients were divided into a pneumonia group and a non-pneumonia group. Baseline characteristics for the two groups are presented in Table 1. The results indicated that patients in the pneumonia group had higher Fisher and Hunt-Hess scale scores (*p* < 0.01). Furthermore, the pneumonia group exhibited a higher prevalence of dysphagia, cerebral cardiac syndrome, hydrocephalus (*p* < 0.01), and hypertension (*p* = 0.05). Regarding blood parameters, patients in the pneumonia group had significantly higher levels of neutrophils (*p* < 0.001) and white blood cells (*p* < 0.001). In addition, the pneumonia group showed elevated biomarker levels compared to the non-pneumonia group: SIRI: Median (IQR) 5.00 (2.75–8.44) vs. 3.62 (2.08–6.70), *p* < 0.01. SII: Median (IQR) 2,374.27 (1,429.67–3,478.80) vs. 1,810.71 (1,031.62–3,042.18), *p* < 0.01. NLR: Median (IQR) 11.86 (6.92–15.80) vs. 8.36 (4.68–13.90), *p* < 0.01. LDH: Median (IQR) 262.00 (217.00–289.00) vs. 183.50 (156.00–216.00), *p* < 0.01. These findings highlight significant differences in clinical and blood-based parameters between the pneumonia and non-pneumonia groups.

**Table 1:** Baseline characteristics of aneurysmal SAH patients who received operative treatment with pneumonia and non-pneumonia.

Variable	Total (*n* = 1,049)	Non-pneumonia (*n* = 823)	Pneumonia (*n* = 226)	*p*
Age, median(IQR), year	58.00(51.00, 66.00)	57.00(50.00, 65.00)	60.00(53.00, 67.00)	0.007
Sex, female, n(%)	642 (61.2)	501 (60.9)	141 (62.4)	0.736
BMI, median(IQR), year	20.60(18.90, 23.10)	20.50(18.90, 23.10)	20.75(18.90, 23.20)	0.125
Past medical history				
Hypertension, n(%)	611 (58.2)	461 (56.0)	150 (66.4)	0.005
Diabetes, n(%)	94 (9.0)	68 (8.3)	26 (11.5)	0.131
CHD, n(%)	98 (9.3)	77 (9.4)	21 (9.3)	0.977
CI, n(%)	73 (7.0)	55 (6.7)	18 (8.0)	0.503
Hyperlipidemia, n(%)	15 (1.4)	12 (1.5)	3 (1.3)	0.884
Previous or current smoking, n(%)	445 (42.4)	371 (45.1)	74 (32.7)	0.001
Previous or current drinking, n(%)	282 (26.9)	237 (28.8)	45 (19.9)	0.006
Admission vital signs				
Respiration rate breaths/ min	18.00 (16.00, 19.00)	18.00(16.00, 19.00)	17.00(16.00, 19.00)	0.709
MAP, mm Hg	88.00(79.00, 93.00)	88.00(79.00, 93.00)	88.50(80.00, 93.00)	0.147
Heart rate/min	88.00(74.00, 98.00)	87.00(74.00, 98.00)	88.00(74.00, 98.00)	0.665
Dysphagia, n(%)	132 (12.6)	58 (7.1)	74 (32.7)	<0.001
MV, n(%)	38 (3.6)	10 (1.2)	28 (12.4)	<0.001
Modified Fisher scale				
1–2	791 (75.4)	683 (83.0)	108 (47.8)	0.543
3–4	258 (24.6)	146 (17.7)	112 (49.6)	<0.001
HUNT-HESS scale				
I-II	924 (88.1)	769 (93.4)	155 (68.6)	0.437
III-V	125 (11.9)	54 (6.6)	71 (31.4)	<0.001
Laboratory indicators				
Neutrophil, median(IQR), 10^9^/L	9.50(7.10, 12.50)	9.10(6.70, 11.80)	11.15(8.22, 14.10)	<0.001
WBC, median(IQR), 10^9^/L	11.20(9.10, 13.90)	10.80(8.70, 13.40)	13.10(10.10, 16.10)	<0.001
Lymphocytes, median(IQR), 10^9^/L	1.10(0.80, 1.60)	1.10(0.80, 1.60)	1.00(0.70, 1.40)	0.054
Monocytes, median(IQR), 10^9^/L	0.50(0.30, 0.70)	0.50(0.40, 0.70)	0.50(0.30, 0.70)	0.573
Platelets, median(IQR), 10^9^/L	222.00(188.00, 261.00)	221.00(188.00, 262.00)	224.00(185.50, 260.00)	0.886
RBC, median(IQR), 10^9^/L	4.39(4.04, 4.74)	4.40(4.08, 4.73)	4.32(3.85, 4.81)	0.059
Albumin, median(IQR), g/L	41.10(38.45, 43.90)	41.10(38.45, 43.90)	41.50(38.45, 44.12)	0.543
CRP, median(IQR), mg/L	6.70(2.10, 17.74)	5.70(1.60, 13.40)	16.15(5.14, 45.84)	<0.001
NLR, median(IQR)	9.00(5.07, 14.38)	8.36(4.68, 13.90)	11.86(6.92, 15.80)	<0.001
NAR, median(IQR), g	0.23(0.18, 0.30)	0.22(0.17, 0.29)	0.28(0.21, 0.34)	<0.001
MLR, median(IQR)	0.42(0.29, 0.64)	0.41(0.29, 0.64)	0.44(0.33, 0.67)	0.016
SIRI, median(IQR), 10^9^/L	3.98(2.16, 6.94)	3.62(2.08, 6.70)	5.00(2.75, 8.44)	<0.001
SII, median(IQR), 10^9^/L	1923.64(1094.80, 3150.00)	1810.71(1031.62, 3042.18)	2374.27(1429.67, 3478.80)	<0.001
AISI, median(IQR), 10^27^/L	855.27(452.93, 1577.95)	826.57(428.82, 1473.84)	1163.56(541.42, 2189.66)	<0.001
CLR, median(IQR), g	6.06(1.70, 18.30)	5.17(1.30, 13.26)	16.18(3.60, 56.03)	<0.001
FAR, median(IQR)	0.08(0.07, 0.09)	0.08(0.07, 0.09)	0.08(0.07, 0.09)	0.18
PLR, median(IQR), 10^9^/L	195.00(137.33, 294.00)	190.59(135.33, 297.56)	211.25(151.43, 291.35)	0.063
PNI, median(IQR)	47.10(44.40, 50.60)	47.20(44.50, 50.50)	47.10(43.90, 50.77)	0.46
LDH, median(IQR), U/L	191.50(163.00, 240.00)	183.50(156.00, 216.00)	262.00(217.00, 289.00)	<0.001
ALT, median(IQR), U/L	16.23(12.22, 24.03)	16.00(12.05, 23.96)	17.26(13.04, 24.00)	0.09
AST, median(IQR), U/L	20.03(15.78, 26.62)	19.46(15.52, 25.13)	23.02(16.76, 31.26)	<0.001
Total bilirubin, median (IQR), umol/L	9.90(7.60, 13.30)	10.00(7.65, 13.40)	9.50(7.50, 12.85)	0.389
Direct bilirubin, median (IQR), umol/L	2.80(2.10, 3.80)	2.70(2.10, 3.80)	2.80(2.10, 3.70)	0.456
Urea, median(IQR), umol/L	4.34(3.52, 5.35)	4.29(3.48, 5.22)	4.61(3.72, 5.93)	0.001
Creatinine, median(IQR), umol/L	54.80(46.63, 63.60)	55.00(46.55, 64.04)	54.20(46.93, 62.66)	0.847
Troponin I, median(IQR), ng/L	0.01(0.00, 0.01)	0.01(0.00, 0.01)	0.01(0.01, 0.04)	<0.001
Troponin T, median(IQR), U/L	10.00(7.00, 14.00)	10.00(7.00, 13.00)	12.00(8.00, 21.00)	<0.001
B-type natriuretic peptide, median(IQR), pg./mL	45.60(34.17, 90.24)	45.23(32.36, 74.22)	89.40(39.89, 729.85)	<0.001
Thrombin time, median (IQR), s	13.20(12.70, 13.60)	13.10(12.70, 13.60)	13.20(12.80, 13.60)	0.142
Activated clotting time, median(IQR), s	32.90(30.50, 35.20)	33.10(30.70, 35.25)	32.20(29.90, 34.92)	0.095
D-dimer, median(IQR), mg/L	1.49(0.81, 2.88)	1.33(0.76, 2.41)	2.31(1.19, 5.05)	<0.001
Anticoagulant, median(IQR), U/L	100.00(92.00, 108.00)	101.00(91.00, 108.00)	100.00(94.00, 108.00)	0.571
Fibrinogen, median(IQR), g/L	3.32(2.86, 3.78)	3.33(2.89, 3.77)	3.25(2.75, 3.78)	0.242
Plasma fibrin degradation product, median(IQR), ug/mL	4.67(2.65, 12.77)	4.25(2.39, 9.87)	8.29(3.81, 24.06)	<0.001
Glucose, median(IQR), mmol/L	7.07(6.10, 8.73)	6.81(6.00, 8.27)	8.34(6.91, 9.86)	<0.001
Hemoglobin, median(IQR), g/L	134.00(123.00, 143.00)	134.00(124.00, 144.00)	130.50(118.00, 142.00)	0.004
Sodium ion, median(IQR), mmol/L	137.00(135.00, 139.00)	137.00(135.00, 139.00)	137.00(135.00, 139.00)	0.571
Potassium ion, median (IQR), mmol/L	3.74(3.50, 3.96)	3.74(3.50, 3.96)	3.74(3.49, 3.99)	0.68
Chloride ion, median(IQR), mmol/L	102.90(100.80, 105.20)	102.90(100.80, 105.10)	103.00(101.00, 105.40)	0.393
Location, n(%)				
ICA, n(%)	448 (0.43)	353(0.43)	95 (0.42)	0.751
MCA, n(%)	203 (19.4)	153 (18.6)	50 (22.1)	0.234
AcomA, n(%)	363 (34.6)	293 (35.6)	70 (31.0)	0.196
V-BA, n(%)	35 (3.3)	24 (2.9)	11 (4.9)	0.148
Operation, n(%)				
Aneurysm clipping, n(%)	525 (50.0)	401 (48.7)	124 (54.9)	0.134
Endovascular intervention, n(%)	524(50.0)	436(53.0)	88(38.9)	0.102
Postoperative complications, n(%)				
DCI, n(%)	145(0.14)	56(0.07)	89(0.39)	0.011
CCS, n(%)	187 (17.8)	101 (12.3)	86 (38.1)	<0.001
HCP, n(%)	75 (7.2)	30 (3.7)	45 (19.9)	<0.001
EP, n(%)	8 (0.8)	8 (1.0)	0 (0.0)	0.137

IQR, interquartile range; BMI, body mass index; CHD, coronary heart disease; CI, cerebral infarction; MAP, mean arterial pressure; MV, mechanical ventilation; WBC, white blood cell; RBC, red blood cell; CRP, c-reactive protein; NLR, neutrophil-to-lymphocyte ratio; NAR, neutrophil-to-albumin ratio; MLR, monocyte-to-lympholyte ratio; SIRI, systemic infammatory response index; SII, systemic immune infammation index; AISI, aggregate index of systemic inflammation; CLR, c-reactive protein-to-lymphocyte ratio; FAR, fibrinogen-to-albumin ratio; PLR, platelet-to-lymphocyte ratio; PNI, prognostic nutritional index; LDH, lactate dehydrogenase; ALT, alanine aminotransferase; AST, aspartate aminotransferase; ICA, internal carotid artery; MCA, middle cerebral artery; AcomA, anterior communicating artery; V-BA, vertebrobasilar artery; DCI, delayed cerebral ischemia; CCS, cerebral cardiac syndrome; HCP, hydrocephalus; EP, epilepsy. NLR, neutrophil count/lymphocyte conut; NAR, neutrophil count/albumin; MLR, monocyte count/lympholyte count; PLR, platelet count/lymphocyte count; SIRI, monocyte count×neutrophil count/lymphocyte count; SII, platelet count×neutrophil count/lymphocyte count; CLR, c-reactive protein/lymphocyte count; FAR, fibrinogen/albumin; PLR, platelet count/lymphocyte count; PNI, albumin+5 × lymphocyte count.

### 3.2 Multivariate logistic regression analysis

All variables were initially subjected to univariate logistic analysis, and those with *p* < 0.05 were included in the multivariate logistic analysis. In the multivariate logistic regression analysis, significant differences were observed in white blood cells, neutrophils, LDH, NLR, MLR, SIRS, and SII (*p* < 0.05; Table 2).

**Table 2:** Multivariate analysis.

Variable	Multivariate analysis	
	OR(95%CI)	*p*
Age, median(IQR), year	0.980(0.962–0.999)	0.123
Hypertension, n(%)	1.333(0.865–2.053)	0.193
Previous or current drinking, n(%)	0.667(0.082–5.457)	0.706
Dysphagia, n(%)	1.009(0.479–2.124)	0.982
MV, n(%)	2.860(0.879–9.305)	0.081
Modified Fisher scale(III-IV)	1.419(0.841–2.391)	0.543
HUNT-HESS scale(III-IV)	1.489(0.796–2.795)	0.437
Neutrophil, median(IQR), 10^9^/L	1.165(0.883–1.537)	**0.024**
WBC, median(IQR), 10^9^/L	0.805(0.638–1.015)	**0.031**
CRP, median(IQR), mg/L	1.015(0.999–1.031)	0.067
NLR, median(IQR)	0.890(0.807–0.983)	**0.025**
NAR, median(IQR), g	0.719(0.010–53.411)	0.881
MLR, median(IQR)	5.979(1.335–26.779)	**0.019**
SIRI, median(IQR), 10^9^/L	1.182(1.001–1.395)	**0.048**
SII, median(IQR), 10^9^/L	1.089(0.935–1.115)	**0.028**
AISI, median(IQR), 10^27^/L	1.121(1.079–1.540)	0.598
CLR, median(IQR), g	1.291(1.080–1.561)	0.088
LDH, median(IQR), U/L	0.984(0.980–0.988)	**<0.001**
AST, median(IQR), U/L	1.005(0.996–1.014)	0.321
Urea, median(IQR), umol/L	1.013(0.897–1.144)	0.834
Troponin I, median(IQR), ng/L	7.462(0.805–69.169)	0.0769
Troponin T, median(IQR), U/L	1.009(0.979–1.040)	0.557
B-type natriuretic peptide, median(IQR), pg./mL	0.999(0.956–1.243)	0.762
D-dimer, median(IQR),mg/L	1.007(0.954–1.063)	0.807
plasma fibrin degradation product, median(IQR), ug/mL	0.991(0.980–1.001)	0.088
glucose, median(IQR), mmol/L	1.000(0.913–1.095)	0.996
hemoglobin, median(IQR),g/L	1.056(0.918–1.156)	0.261
DCI, n(%)	0.906(0.883–1.068)	0.784
CCS, n(%)	0.967(0.832–1.134)	0.867
HCP, n(%)	0.833(0.811–0.999)	0.995

Bold values indicate *p*-values less than 0.05.

To account for potential confounding effects, we utilized covariate-adjusted logistic regression and conducted a comprehensive quartile-based analysis. Two models were applied to assess significant inflammatory markers: an unadjusted model and a multivariate logistic regression model incorporating all relevant confounders. Table 3 provides comprehensive assessments of multiple inflammatory markers (LDH, SIRI, SII, NLR, and MLR) in the quartiles (Q1-Q4) for both the unadjusted model (Model 1) and the adjusted model (Model 2), including the odds ratio (OR), 95% confidence interval (limits), and *p*-values. For most biomarkers, the high quartile in the unadjusted model shows a higher OR and lower p-value, suggesting a possible relationship. However, these relationships are often weakened in the adjusted model. Notably, LDH maintained a strong association in both models, particularly in Q4 with an extremely high OR and a very low adjusted *p*-value. The OR in Q4 was extremely high in the unadjusted Model 1, at 28.49, with a p-value <0.0001. Even in the adjusted Model 2, the OR in Q3 was still high, at 6.69, and in Q4 it was 30.28, both with *p*-values <0.0001; this shows a significantly strong relationship between high LDH quartiles and the outcomes. For SIRI, in the unadjusted Model 1, the OR from Q1-Q4 increased significantly as the quartile increased (0.76–1.33), with *p*-values increasing from greater than 0.05 to less than 0.01. In contrast, the OR in the adjusted Model 2 increased as the quartile increased. For SII, in Model 1, the OR in Q2 was 1.61, while in Q3 and Q4, the OR significantly increased to 2.53 and 2.65, respectively, with both p-values <0.0001. However, the trend was reversed in the adjusted Model 2. Other biomarkers (NLR and MLR) demonstrated similar trends, i.e., in the unadjusted Model 1, high quartiles showed an increasing OR and lower *p*-value, suggesting a significantly strong relationship. However, these relationships weakened in the adjusted Model 2.

**Table 3:** The quartile of blood-based biomarkers in patients with aSAH who received operative treatment and the risk of aSAH-associated pneumonia.

Variable	Model	Quartile	OR	CI_Lower	CI_Upper	p_value
SIRI	Unadjusted	2	1.21	0.761	1.924	0.421
		3	1.966	1.271	3.039	0.002
		4	2.056	1.332	3.173	0.001
	adjusted	2	0.82	0.475	1.417	0.477
		3	0.735	0.404	1.336	0.312
		4	0.311	0.142	0.685	0.004
SII	Unadjusted	2	1.605	0.994	2.593	0.053
		3	2.534	1.605	4.001	<0.0001
		4	2.649	1.68	4.175	<0.0001
	adjusted	2	0.918	0.514	1.637	0.771
		3	0.933	0.498	1.748	0.828
		4	0.415	0.187	0.922	0.031
NLR	Unadjusted	2	1.487	0.921	2.402	0.105
		3	2.592	1.652	4.068	<0.0001
		4	2.486	1.58	3.91	<0.0001
	adjusted	2	0.589	0.329	1.054	0.075
		3	0.658	0.356	1.218	0.183
		4	0.268	0.124	0.58	0.001
MLR	Unadjusted	2	1.678	1.089	2.586	0.019
		3	1.44	0.928	2.236	0.104
		4	1.681	1.081	2.615	0.021
	adjusted	2	1.46	0.874	2.438	0.148
		3	0.984	0.571	1.696	0.954
		4	0.454	0.238	0.865	0.016
LDH	Unadjusted	2	2.216	1.052	4.67	0.036
		3	5.405	2.741	10.656	<0.0001
		4	28.489	14.851	54.652	<0.0001
	adjusted	2	2.896	1.24	6.763	0.014
		3	6.685	3.02	14.798	<0.0001
		4	30.28	13.733	66.762	<0.0001

### 3.3 Subgroup analysis and sensitivity analysis

To adjust for potential confounding factors and improve the robustness and credibility of the results. We revisited relevant literature and identified key factors associated with pneumonia following acute brain injury, including age, dysphagia, Fisher grade, Hunt-Hess grade, mechanical ventilation, and smoking history. Given that these variables were already included as covariates in our model, we conducted subgroup and sensitivity analyses to further validate our results.

**Figure 2:**
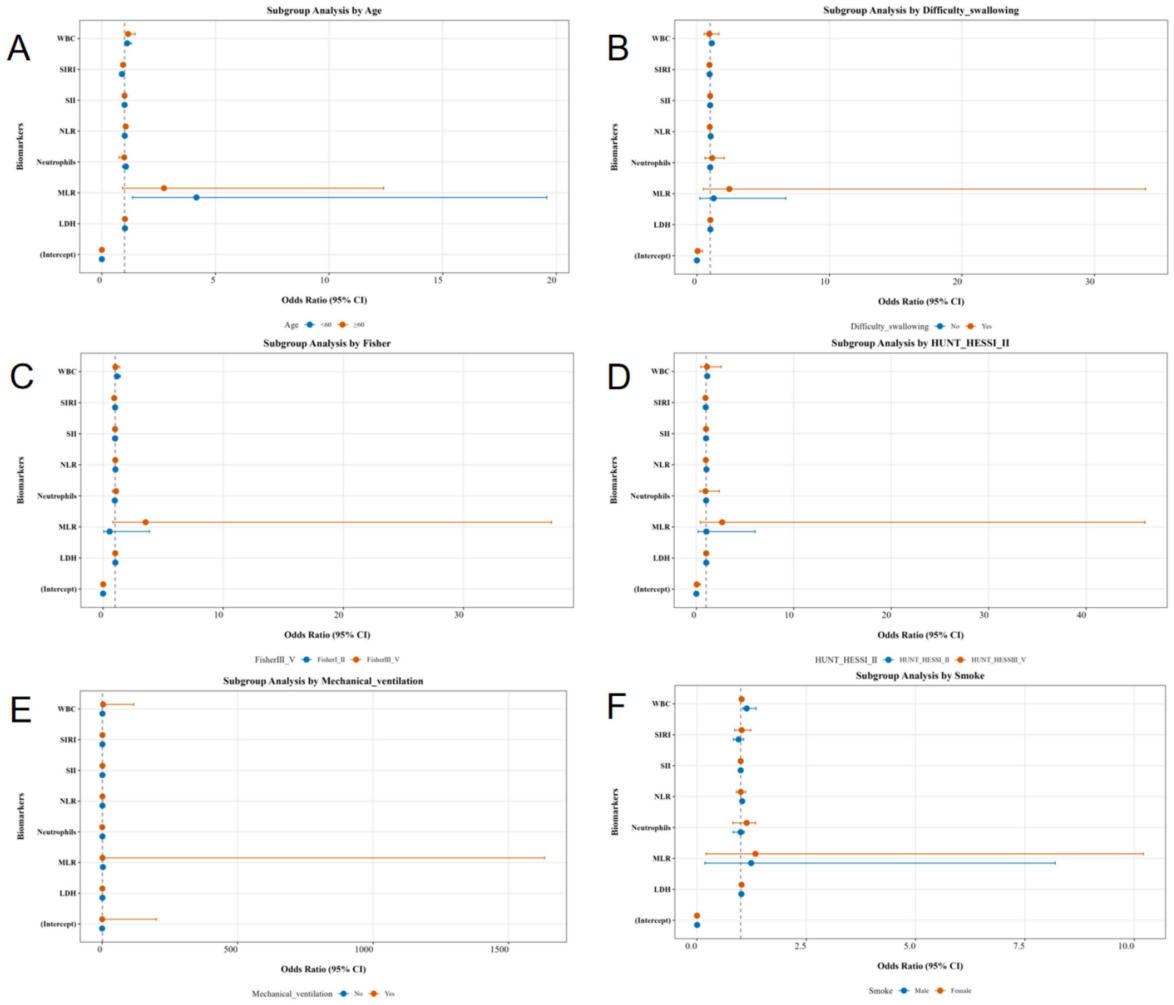
Subgroup analysis of age **(A)**, Difficulty in swallowing **(B)**, Fisher **(C)**, Hunt-Hess **(D)**, Mechanical ventilation **(E)**, Smoke **(F)**.

Subgroup Analysis: Stratified binary logistic regression models were developed to assess effect variations of individual indicators—including age, dysphagia, Fisher grade, Hunt-Hess grade, mechanical ventilation, and smoking history—across different subgroups. Model construction: pneumonia incidence (Pneumonia) was used as the dependent variable (reference group: no pneumonia), and independent variables included seven indicators (WBC, Neutrophils, NLR, MLR, SII, SIRI, and LDH). Results (Figure 2): after adjusting for confounding factors, no significant differences were observed in the associations of WBC, Neutrophils, NLR, SII, SIRI, or LDH with pneumonia risk across subgroups. The exception was MLR, which exhibited subgroup-specific variability. These findings confirm the robustness and reliability of the results.

**Figure 3:**
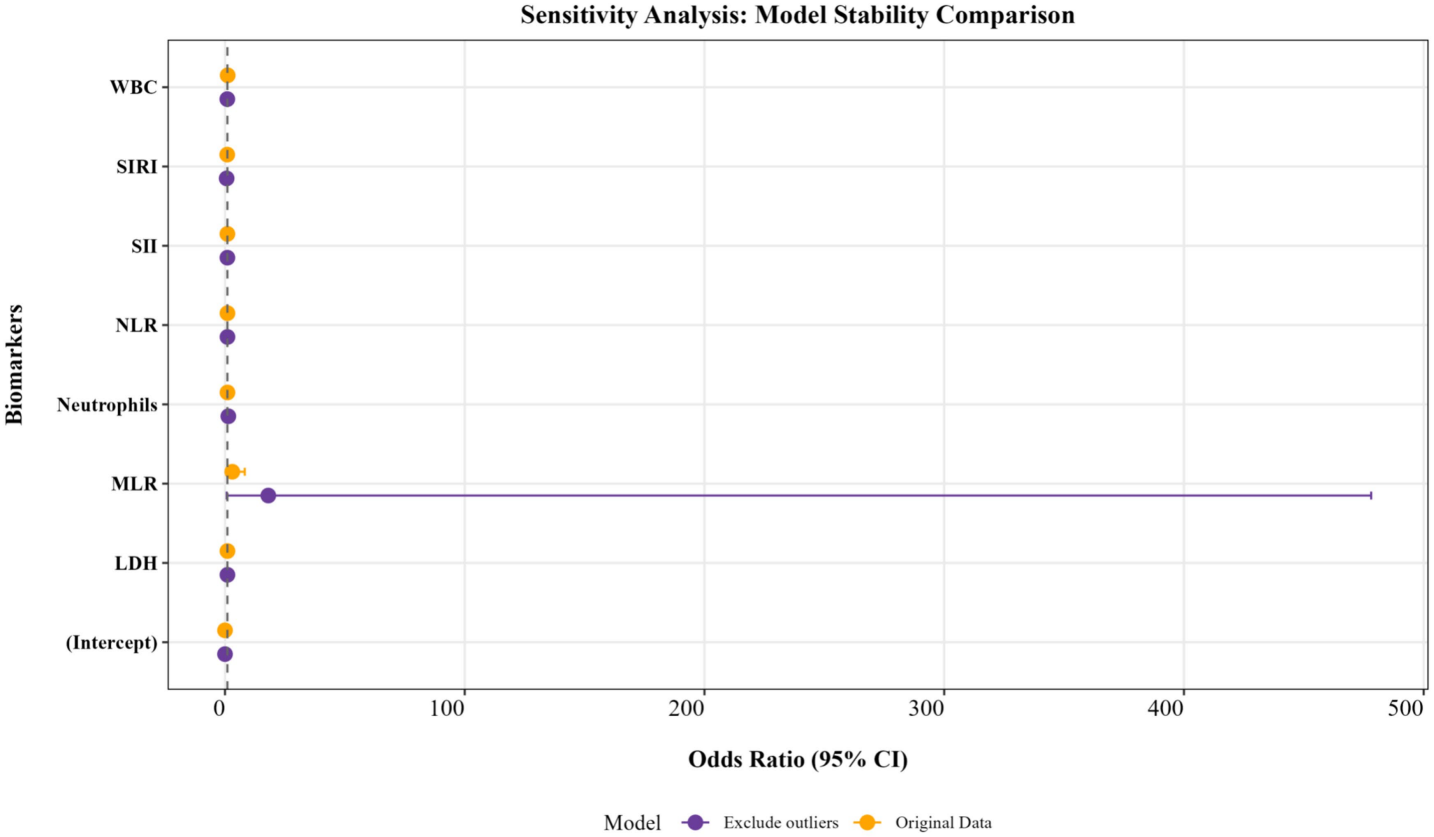
Sensitivity analysis (model stability assessment).

Among these, the sensitivity analysis (model stability assessment) is used to verify the reliability of core results and exclude outliers for seven indicators (WBC, Neutrophils, NLR, MLR, SII, SIRI, and LDH). First, a Logistic regression model (original mode) is constructed based on the raw data. Then, outliers are excluded to obtain the sensitivity analysis dataset, which is re-modeled (sensitivity model). By comparing the OR values, 95% CI, and *p*-values of each variable between the original model and the sensitivity model, the impact of outliers on model coefficients is evaluated. The final analysis results are shown in the figure. The results indicate that, apart from the MLR indicator, there is no significant difference between the two models for WBC, Neutrophils, NLR, S II, SIRI, and LDH indicators, suggesting that the original analysis results are stable and reliable (Figure 3). Outliers were excluded using statistical methods based on interquartile range (interquartile range, IQR). For WBC, Neutrophils, NLR, MLR, SII, SIRI, and LDH, the data distribution range is determined by calculating the first quartile (Q1) and the third quartile (Q3). The threshold for outliers is defined as: lower limit = Q1-1.5 × IQR; upper limit = Q3 + 1.5 × IQR. Extreme values that fall outside this range are identified and recorded.

### 3.4 Receiver operating characteristic curve analysis

After comparing the predictive performance of LDH with SIRI, SII, NLR, and MLR, the AUC of LDH (0.8148, 95% CI: 0.7829–0.8468) was significantly higher than that of SIRI (AUC: 0.5994, 95% CI: 0.5584–0.6414), SII (AUC: 0.5950, 95% CI: 0.5548–0.6352), and NLR (AUC: 0.6064, 95% CI: 0.5667–0.646). These findings further emphasize the superior predictive accuracy of LDH than other biomarkers. The optimal cut-off value for LDH to predict pneumonia was 1.545, with a sensitivity of 73% and a specificity of 81.5% (Table 4).

**Table 4:** The predictive ability of blood-based biomarkers for pneumonia.

Biomarker	AUC	CI_Lower	CI_Upper	Sensitivity	Specificity	Youden_Index
LDH-MLR	0.815	0.784	0.847	0.73	0.815	1.545
LDH-SIRI	0.817	0.785	0.849	0.735	0.809	1.544
LDH-SII	0.607	0.567	0.646	0.633	0.561	1.194
LDH-NLR	0.814	0.782	0.846	0.735	0.802	1.536
LDH	0.815	0.783	0.847	0.73	0.815	1.545
SIRI	0.6	0.558	0.641	0.597	0.587	1.184
SII	0.595	0.555	0.635	0.584	0.593	1.177
NLR	0.606	0.567	0.646	0.535	0.661	1.196
MLR	0.534	0.493	0.575	0.642	0.453	1.095

**Figure 4:**
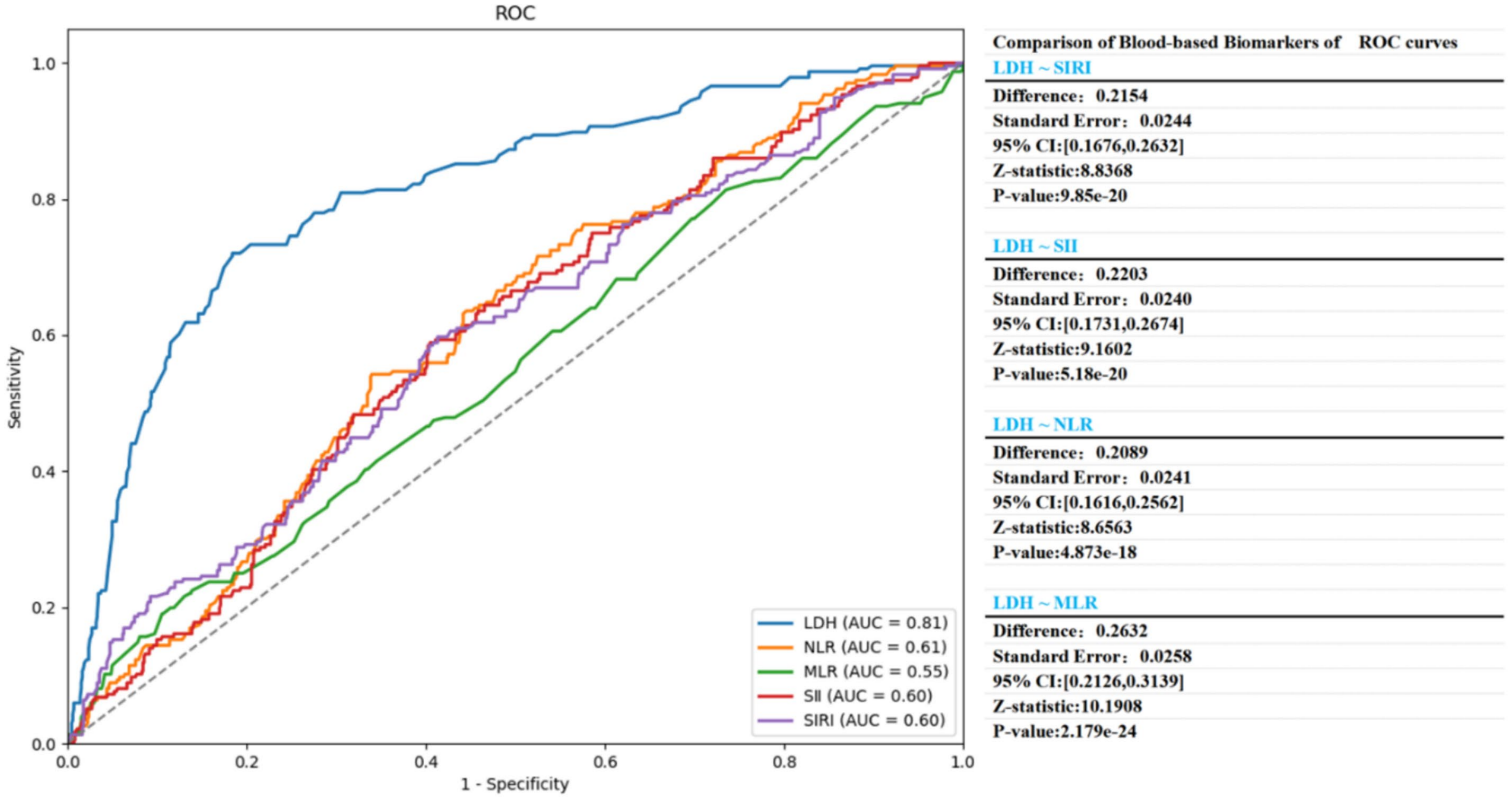
Comparison for the predictive ability of blood-based biomarkers for aSAH-associated pneumonia. ^a^DeLong et al.

**Figure 5:**
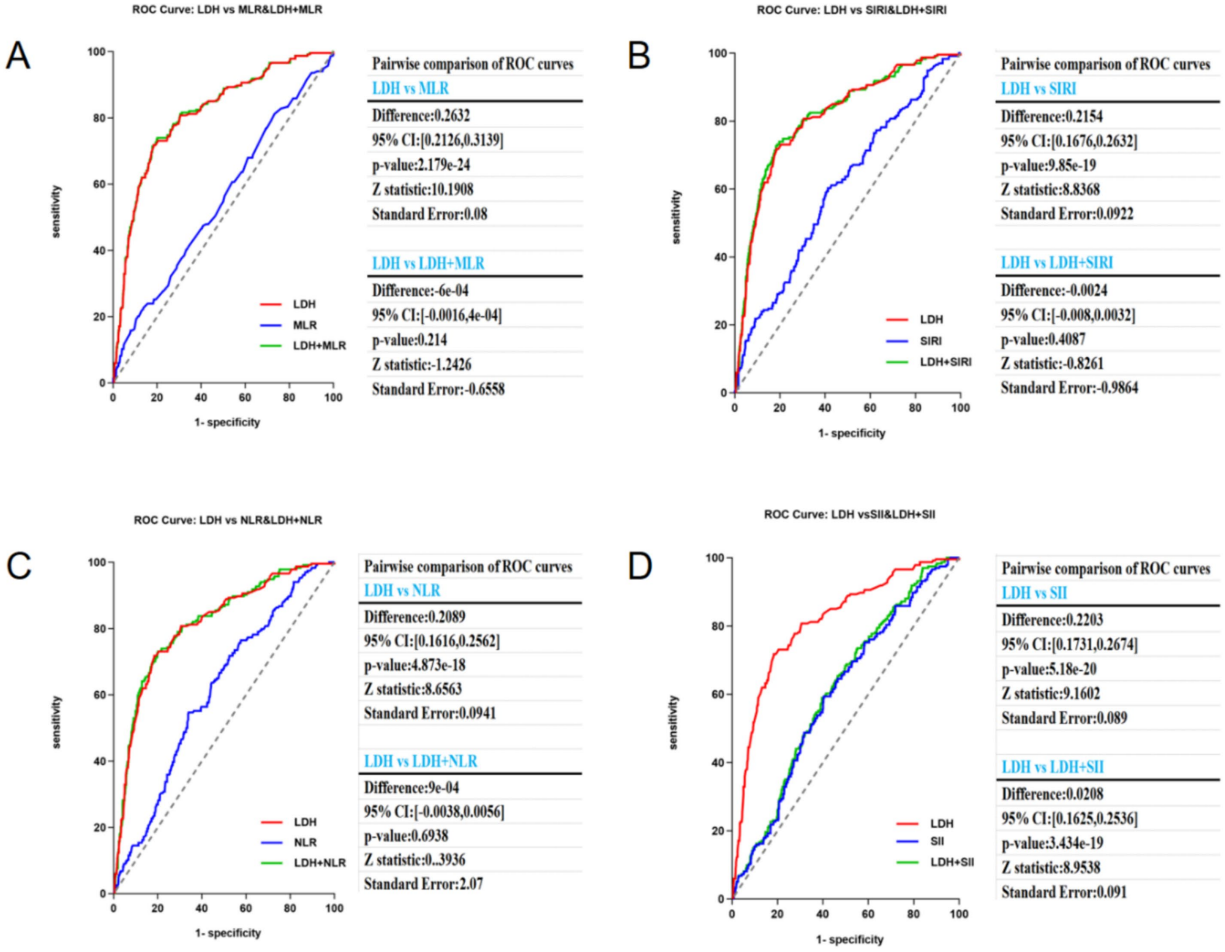
The Predictive ability of LDH, MLR, SIRI, NLR and SII for aSAH-associated pneumonia. **(A)** LDH vs MLR & LDH+MLR ROC curve; **(B)** LDH vs SIRI & LDH+SIRI ROC curve; **(C)** LDH vs NLR & LDH+NLR ROC curve; **(D)** LDH vs SII & LDH+SII ROC curve.

Figure 4 uses ROC curves to assess the predictive performance of LDH alone and combined with other inflammation markers including MLR, SIRI, SII, and NLR, using DeLong’s test to statistically compare the AUC differences. The results revealed the predictive potential of different combinations and their statistical significance. LDH demonstrated high predictive capacity in all figure, with its AUC generally higher than that of individual inflammation markers including NLR. LDH + MLR (Figure 5A): The combination shows a significant improvement (AUC increased to 0.8148). LDH + SIRI (Figure 5B): The combination of LDH with SIRI shows an AUC similar to that of LDH alone, substantially increasing its AUC. LDH + NLR (Figure 5C): The use of NLR + LDH significantly improved its AUC. The combined variables demonstrated improved performance. LDH + SII (Figure 5D): SII alone had a lower predictive capacity (AUC below LDH), and its combination with LDH did not improve its AUC. This analysis emphasizes the excellent predictive capacity of LDH when used alone and its different states when combined with other markers. The statistical significance of these combinations hinged on the specific marker paired with LDH.

### 3.5 Restricted cubic spline analysis

**Figure 6:**
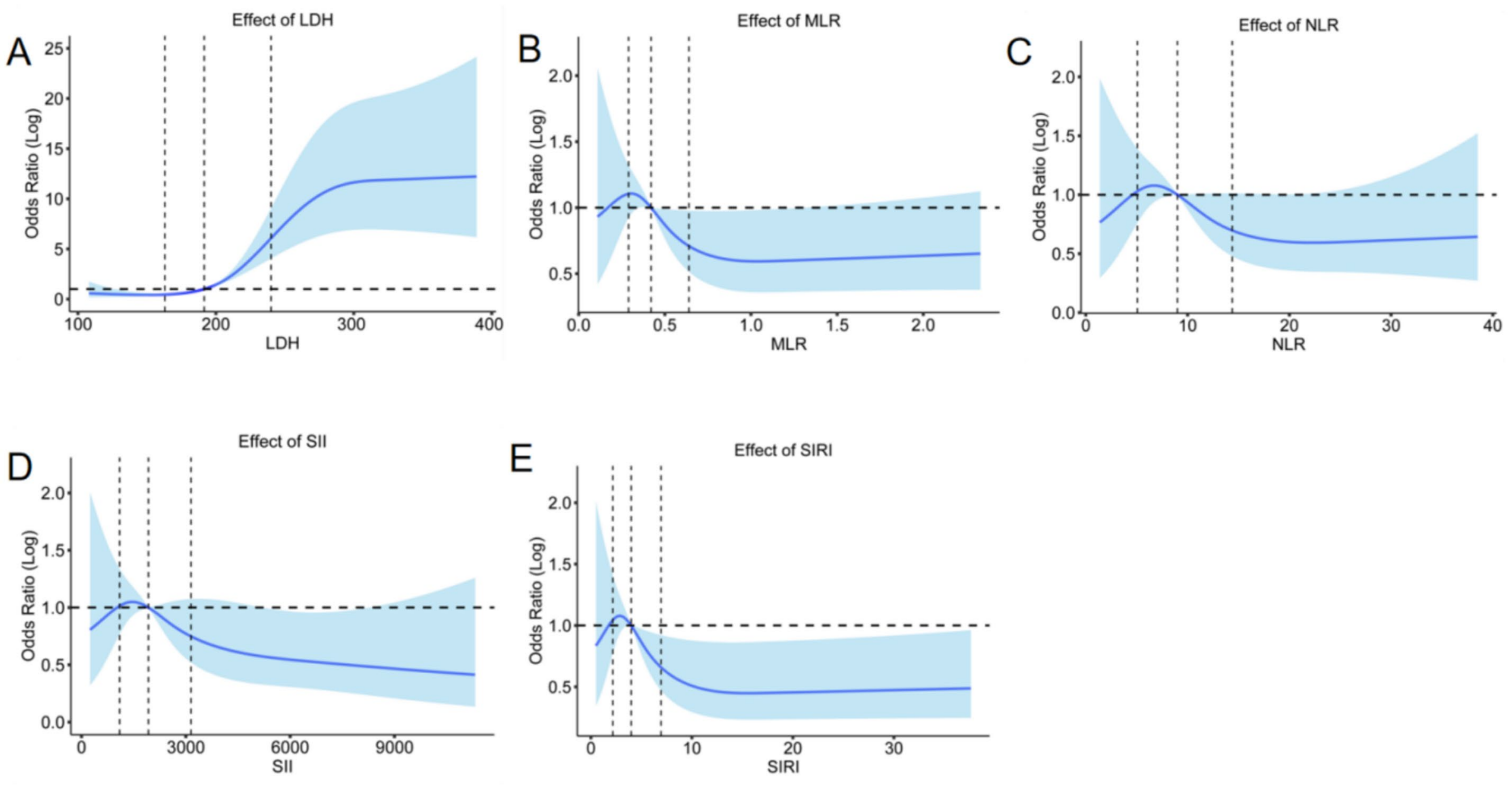
Adjusted odds ratios of blood-based biomarkers of LDH **(A)**, MLR **(B)**, NLR **(C)**, SII **(D)** and SIRI **(E)** with aSAH-associated pneumonia.

To account for potential nonlinear relationships between inflammatory markers and subarachnoid hemorrhage, restricted cubic spline analysis was introduced. Figures 6A–E illustrate the nonlinear associations of LDH, MLR, NLR, SII, and SIRI with an increased risk of pneumonia, respectively. A critical observation is highlighted in Figure 4A: when LDH levels remain below 200 U/L, the pneumonia risk remains relatively low, but once LDH exceeds 200 U/L, the risk escalates sharply. This threshold effect and the marked nonlinearity suggest that LDH exerts significantly amplified adverse effects on clinical outcomes beyond specific thresholds, underscoring a positive correlation between elevated LDH levels and pneumonia occurrence. Figures 6A–E show the non-linear relationship between LDH, MLR, NLR, SII, and SIRI and the risk of pneumonia, analyzed by restricted cubic splines (RCS). LDH was linked to the risk of pneumonia.

### 3.6 Construction and evaluation of nomogram

**Figure 7:**
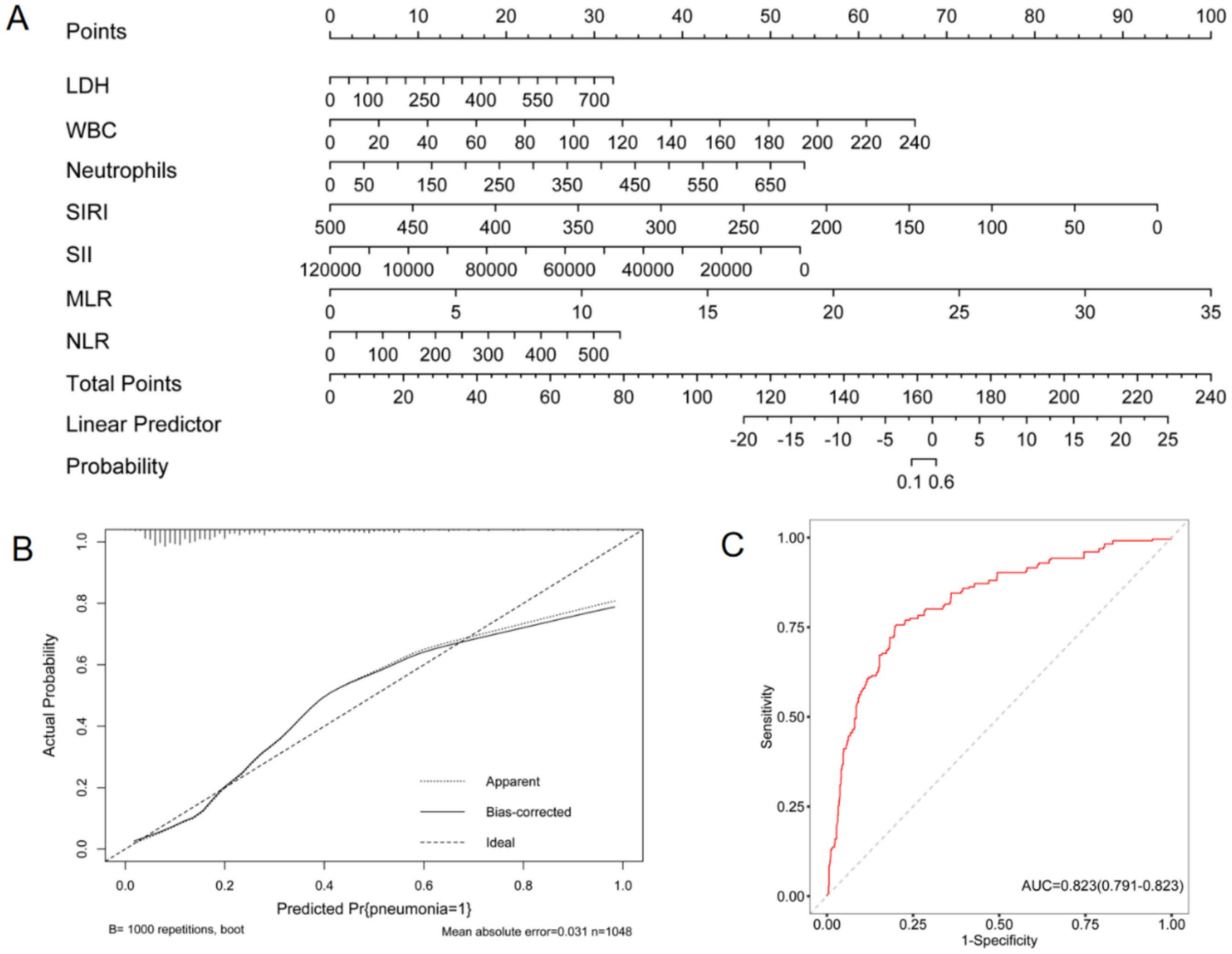
Utility of nomogram for predicting pneumonia after aneurysmal subarachnoid hemorrhage by surgical treatment. Points were LDH, WBC, Neutrophils, SIRI, SII, NLR, MLR. **(A)**, Nomogram **(B)**, Calibration curve **(C)**, Roc curve.

Relevant risk factors derived from the multivariate logistic regression analysis were incorporated into the Receiver Operating Characteristic (ROC) curve to better predict the risk of developing pneumonia (Figure 7A). Based on the logistic regression analysis results described above, a nomogram for predicting pneumonia in aSAH patients was constructed using the R package “rms.” The results demonstrated the following scoring increments in the nomogram prediction model: LDH: 7 points per 150 U/L increase; White blood cells: 6 points per 20 × 10^9^/L increase; Neutrophils: 7 points per 100 × 10^9^/L increase; SIRI: 10 points per 50 × 10^9^/L decrease; SII: 9 points per 20,000 × 10^9^/L decrease; NLR: 14 points per 5-unit increase; MLR: 6 points per 100-unit increase. Increased scores are associated with a higher risk of pneumonia. The calibration curve (Figure 7B) shows a strong agreement between the predicted and observed probabilities. Furthermore, the C index was 0.82, and the ROC curve (Figure 7C) showed excellent discrimination. The area under the ROC curve (AUC) for predicting pneumonia using the ROC curve was 0.82 (95% CI 0.791—0.823; *p* < 0.001).

### 3.7 Internal validation

**Figure 8:**
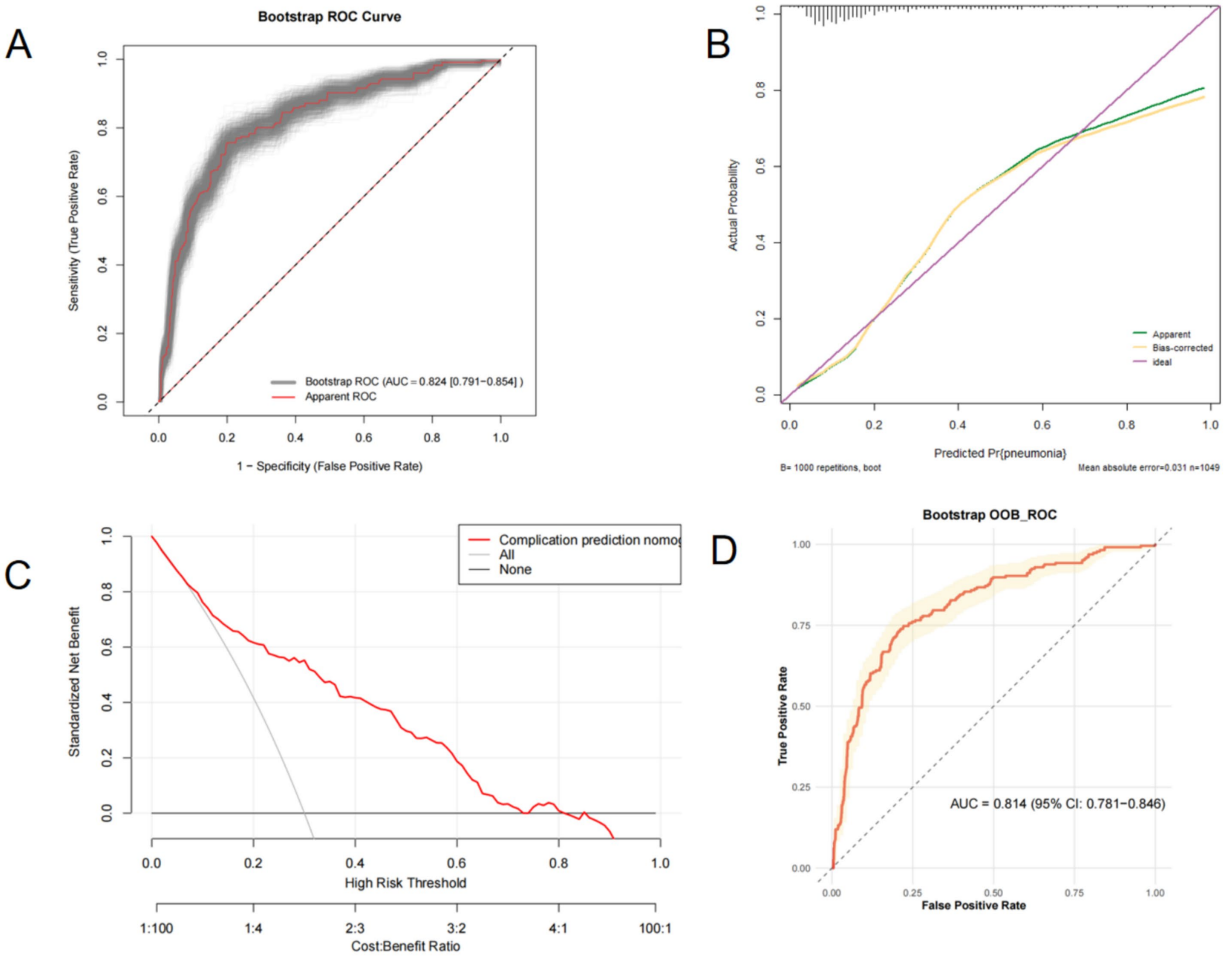
Internal validation of the nomogram was conducted via the bootstrap method. **(A)**, Boostrap ROC curve **(B)**, Calibration curve **(C)**, Decision curve **(D)**, Boostrap OOB ROC curve.

In this study, we performed internal validation using the bootstrap method. The ROC curve demonstrates the model’s stability, with an average AUC and confidence interval of 0.824 (95% CI: 0.791–0.854). An AUC > 0.75 indicates strong discriminative ability of the model for pneumonia risk, and the narrow confidence interval suggests robust and reliable results (Figure 8A). Furthermore, calibration curves with 1,000 bootstrap resamples reveal relatively stable model performance, though its predictive ability appears moderate (Figure 8B). Additionally, decision curve analysis with 1,000 bootstrap iterations shows that the model provides net benefit across the threshold probability range of 0–70% (Figure 8C). The study demonstrated that the model achieved an area under the ROC curve (AUC) of 0.814 (95% CI: 0.781–0.846) through Bootstrap OOB validation, indicating robust discriminative ability to reliably stratify pneumonia risk. No significant overfitting was observed during the validation process, as evidenced by the narrow confidence interval width (6.5%) of the OOB-validated AUC that did not cross the 0.8 threshold. These findings confirm the model’s generalizability, supported by repeated testing with independent samples through the OOB validation framework (Figure 8D).

## 4 Discussion

Postoperative aSAH-related pneumonia is linked to poor prognosis outcomes. Despite many studies on inflammatory markers related to aSAH (18, 22–25), there is still a lack of systematic review of inflammatory markers. Previous studies have demonstrated the predictive efficacy of inflammatory markers (LDH, SIRI, SII, NLR, MLR) for associated pneumonia following aneurysmal subarachnoid hemorrhage, yet lacked comprehensive analysis of these biomarkers. This study is the first to identify that LDH exhibits stronger predictive performance compared to other inflammatory markers. Previous studies may have certain limitations in statistical analysis. In this study, considering the potential nonlinear relationship between inflammatory markers and the occurrence of pneumonia associated with aneurysmal subarachnoid hemorrhage, we introduced restricted cubic spline analysis—a novel methodological approach not previously reported in earlier similar research. To control for confounding factors, we incorporated stratified analysis and sensitivity analysis into our statistical methodology—a comprehensive approach that had not been employed in previous similar studies. Herein, LDH, NLR, SIRI, SII, and MLR were analyzed using multivariate logistic regression analysis and ROC curve analysis, DeLong’s test, restricted independent sample t-test, as well as the analysis of the independent and combined correlations of inflammatory markers. We constructed a nomogram to predict the occurrence of aSAH-related postoperative pneumonia. For the first time, this work shows the relationship between LDH and NLR, SIRI, SII, and MLR, with LDH having a higher predictive accuracy than other biomarkers. This confirms that LDH is significantly associated with the occurrence of pneumonia following aSAH, with excellent individual predictive capacity. Multivariate analysis for the first time also showed that the combination of LDH and MLR, LDH and SIRI, and LDH and NLR can increase the predictive capacity; however, LDH and SII do not have similar effects. The risk prediction by the nomogram is highly consistent with the observed probability, revealing excellent predictive capacity for the occurrence of aSAH-related postoperative pneumonia.

In this study, we found that the incidence of pneumonia in aSAH patients was 21.5%, which was consistent with that reported previously ranging from 13.2 to 30.8% (17–21). Moreover, seven risk factors of pneumonia in aSAH patients were identified, including WBC, neutrophil count, LDH, SIRI, SII, NLR, and MLR. A prediction model comprising these factors had high AUC values (0.823; 95% CI: 0.791–0.855; *p* < 0.001), demonstrating its ability to assist clinicians in effectively evaluating the risk of pneumonia development.

In our study, we found that the white blood cell counts and neutrophil count were significantly higher in the pneumonia group than in the non-pneumonia group. Elevated peripheral blood white blood cell counts and neutrophil count at admission in aSAH patients were identified as risk factors for pneumonia in aSAH patients. Similarly, numerous studies have reported an association between increased neutrophil levels and pneumonia in patients with ischemic stroke (26). As a major regulator of inflammation, white blood cells can not only directly and effectively kill pathogens but also regulate the entire inflammatory response process by secreting cytokines, thereby enhancing the body’s normal anti-infection ability. In the immediate stage after stroke occurrence, the peripheral blood white blood cell counts increase exponentially, mainly due to the elevated production of neutrophils, while the level of lymphocytes in circulation may be significantly decrease (26, 27). Following brain injury, immune cells accumulate at the site of damage, with neutrophils being the first to be recruited through the compromised blood–brain barrier. As the inflammatory response advances, brain tissue edema may worsen, potentially suppressing immune system function (28). Moreover, excessive activation of systemic inflammatory response may trigger immune system suppression, which may be one of the key factors contributing to the occurrence of pneumonia after aSAH. In addition, the aggregation of circulating neutrophils in lung tissue stimulates local inflammation and lung injury (29).

It has been demonstrated that LDH is an important biomarker for monitoring the progression of heart diseases, liver diseases and malignant tumors (26, 30). In pneumonia, several studies have uncovered that LDH is a robust predictor of community-acquired pneumonia, mycoplasma pneumonia and complicated pneumonia (31–33). Regarding neuroinflammation, the LDH levels can predict the degree of neuronal injury, and the prognosis of subarachnoid hemorrhage and intracerebral hemorrhage (including traumatic intracerebral hemorrhage) (34–36). However, studies have shown that the correlation between LDH and postoperative pneumonia after aneurysmal subarachnoid hemorrhage is rare in clinical practice. Here, we found that LDH was an independent risk factor for postoperative pneumonia after aneurysmal subarachnoid hemorrhage, and its prediction results were better compared with that of SIRI, SII, NLR and MLR. The correlation between LDH and postoperative pneumonia after aSAH is likely linked to post-aSAH immunological changes. Studies indicate that nearly 80% of aSAH patients experience an early systemic inflammatory response, characterized by increased release of inflammatory cytokines such as IL-6 and IL-10, exacerbating tissue damage throughout the body (37). In humans, LDH is widely expressed in many organs including the heart, skeletal muscle, lung, among other tissues. Even minor tissue damage can trigger a significant increase in serum LDH levels. Therefore, elevated serum LDH levels in aSAH patients may indirectly indicate lung tissue damage. Zheng et al. uncovered that serum LDH levels may reflect the neurological prognosis status, with its levels found to be significantly increased as the Hunt-Hess and Fisher scores increase (36). Notably, high Hunt-Hess and modified Fisher scores predict good prognosis of postoperative pneumonia after aSAH, but this was not observed in this study. LDH emerged as the central biomarker with the highest predictive accuracy in our study. This phenomenon likely originates from neurogenic mechanisms post-SAH, where subarachnoid hemorrhage activates the sympathetic nervous system, triggering massive catecholamine release. This catecholaminergic surge induces pulmonary vasoconstriction and increases pulmonary capillary permeability, ultimately culminating in neurogenic pulmonary edema. The resultant pulmonary edema and inflammatory cascades cause alveolar epithelial damage, releasing LDH into systemic circulation which further amplifies peripheral LDH elevation through positive feedback mechanisms (38–40).

As an important inflammatory marker, SIRI can predict the functional outcomes after aSAH (41–44). Moreover, SIRI is independently associated with delayed cerebral ischemia following subarachnoid hemorrhage and strongly correlates with pneumonia in ischemic and hemorrhagic stroke (23, 38, 45, 46). Qin confirmed that SIRI reliably predicts pneumonia after endovascular treatment of aneurysmal subarachnoid hemorrhage, aligning with the results of this study (23). However, further prospective studies are needed to determine the direct association between SIRI and the occurrence of pneumonia after aSAH. It has been demonstrated that SII can reflect the inflammatory and immune status of patients. It can employed to monitor tumor patients, with its elevated levels associated with poor prognosis (47, 48). To our knowledge, no study has documented the significance of SII in acute vascular events (including stroke and SAH). Two previous studies demonstrated that elevated SII was associated with adverse outcomes in chronic heart failure and coronary artery disease (49).

In the study by Wang et al., it was found that the NLR on the first and third days after surgery was associated with severe hospital-acquired pneumonia in patients with aneurysmal subarachnoid hemorrhage who underwent clipping or embolization surgery, which is consistent with the results of this study (50). However, there are some differences that should be noted. For instance, the NLR value was dynamically monitored in the previous study. Therefore, in subsequent studies, researchers should perform dynamic detection of inflammatory-related indicators. Previous studies have reported AUC values of 0.707 and 0.671 for NLR in predicting postoperative pneumonia in aSAH patients (18, 51). Similarly, research on pneumonia related to ischemic stroke has shown a significant correlation between NLR and the incidence of pneumonia (52, 53). The above studies indicate that NLR, as an easily obtainable inflammatory indicator, not only reflects the intensity of the body’s inflammatory response but also the balance between neutrophils and lymphocytes and the level of immune regulation during the inflammatory process. Following craniocerebral injury, inflammatory mediators trigger neutrophil proliferation. Simultaneously, in SIDS, lymphocytes undergo transformation and apoptosis due to neurohumoral regulation. This process may contribute to the observed increase in NLR. However, the AUC value of NLR in predicting postoperative pneumonia associated with aneurysmal subarachnoid hemorrhage was relatively low. These differences may be due to factors such as the presence of other diseases in patients, adoption of different treatment strategies, blood sampling times, and varied degrees of brain injury. In future studies, larger-scale prospective studies are needed to further clarify the predictive value of NLR for the occurrence of postoperative pneumonia after aSAH. Evidence indicates that MLR after percutaneous coronary intervention can predict major adverse cardiovascular events in patients with acute cerebral infarction. To our knowledge, few studies have investigated MLR in stroke. In this study, we confirmed that MLR predicted the occurrence of postoperative pneumonia due to aneurysmal subarachnoid hemorrhage.

Previously, TAO et al. found that PLR was an independent predictor of DCI development (54). Moreover, PLR could predict the prognosis of pneumonia after intracerebral hemorrhage (18). However, in this study, PLR showed poor predictive value for the occurrence of pneumonia after aSAH. Notably, PLR is the ratio of platelets to lymphocytes, and thus, whether there are differences in the coagulation effect induced by aSAH and intracerebral hemorrhage need to be further clarified. The pathogenesis of pneumonia after aSAH is not well understood, but studies have suggested the role of the aspiration theory and stroke-induced peripheral immune depression syndrome (39). Therefore, the peripheral immune depression syndrome induced by aSAH may explain the close relationship between SIRI, SII, NLR, MLR and postoperative pneumonia after aSAH.

Our study had several limitations: (1) the predictive capacity of inflammation biomarkers is still limited, and the occurrence of pneumonia is a complex process influenced by multiple factors, including the pathogen, immune status, and the body’s resistance. And the etiology of pneumonia in aSAH patients is indeed multifactorial, encompassing pre-admission pulmonary infections, intraoperative ventilator use during hospitalization, and aspiration-related complications. Our study recognizes the presence of numerous potential confounding factors. To mitigate their influence in future research, we plan to implement stricter inclusion criteria through comprehensive pulmonary imaging examinations and laboratory tests. This approach aims to enhance patient selection rigor and minimize the impact of potential confounding factors on study outcomes. Although inflammation markers can indicate the body’s inflammatory state, they may not fully cover all possible factors. We acknowledge the absence of dynamic/serial measurements of inflammatory biomarkers as a study limitation. Temporal trends in biomarker levels could have offered critical insights into disease progression and therapeutic response, particularly given that inflammatory cascades in aneurysmal subarachnoid hemorrhage (aSAH) often exhibit biphasic patterns (early ischemia vs. delayed immunodysregulation). (2) Our sample size may be relatively small, yet establishing prediction models requires a sufficient sample size. The conclusions of this study are entirely derived from clinical data, rendering them heavily contingent upon the quality of input data. Data quality limitations may directly impact the accuracy of predictive outcomes. Furthermore, the reliance on a single-center cohort inherently limits the generalizability of findings due to potential inadequacies in population representativeness. The analytical framework also exhibits methodological constraints, including an over-reliance on statistical assumptions, which may introduce model bias if underlying assumptions are violated. Importantly, the validation process remains insufficient, particularly regarding the absence of *in vivo* validation. Consequently, experimental validation is imperative to corroborate these predictive conclusions. (3) The lack of external validation studies on the predictive ability of inflammation biomarkers may limit the assessment of their accuracy and stability. The current absence of suitable validation databases stems from multifaceted challenges including inherent data heterogeneity across neurocritical care registries, inter-institutional variability in measurement tools and diagnostic criteria for postoperative complications, methodological complexity in harmonizing multidimensional biomarkers with clinical outcomes, and insufficient granularity in existing datasets for adequate control of residual confounding factors. A suitable database for external validation has not yet been found. Our research group will initiate systematic efforts to identify appropriate external databases through multi-institutional collaboration and data harmonization protocols. Specifically, we intend to validate the nomogram’s performance across: temporal validation using subsequent admission cohorts from our tertiary center, geographical validation through partnerships with NCS-QOD (NeuroPoint Alliance Quality Outcomes Database), and mechanistic validation against the CASCADE consortium’s multi-omics cerebrovascular datasets. This phased validation strategy will incorporate covariate shift analysis and domain adaptation techniques to address inherent heterogeneity. (4) We can not completely exclude confounding factors. Furthermore, this is a retrospective study. This study solely utilized retrospectively collected medical records and imaging data obtained from routine clinical diagnosis and treatment, without administering any additional interventions to patients. The laboratory parameters analyzed in this study represent objective measurements, whereas other clinical variables may inherently carry subjective interpretations due to variability in clinical expertise among physicians. This potential subjectivity could introduce potential biases or limit the generalizability of findings. Future studies should consider prospective multicenter studies.

## 5 Conclusion

In conclusion, LDH is a useful predictor of post-aSAH-related pneumonia. Moreover, the developed prediction model may provide insights for designing preventive antibiotic therapy. Nonetheless, there is a need for further prospective multicenter studies to validate the relationship between LDH and post-aSAH-related pneumonia as well as externally validate the nomogram.

## Data Availability

The raw data supporting the conclusions of this article will be made available by the authors, without undue reservation.
